# A Growing Evil Which Should Be Remedied

**Published:** 1892-06

**Authors:** 


### Editorial.

### A GROWING EVIL WHICH SHOULD BE REMEDIED.

There is an evil that physicians meet with almost daily—the renewal of a prescription by patients or some one they may have given it to, without proper directions from the prescriber.

This is a growing evil that may do incalculable harm to the physician and the person taking the contents of the prescription alike. By the patient renewing the prescription and not returning for observation as to the effects of the remedy, which may need changing, etc., and the patient not deriving the beneficial results he expects, charges it to the physician; also by having his prescriptions hacked around, to be used in some self-made diagnosis either in the patient or his friends. For we meet people every day who have a number of prescriptions in their pockets they have collected from time to time, to be used on such occasions as they think proper, by themselves and friends.

How frequently we are met by the statement from patients that they have been taking Doctor Smith or Jones' medicine a long time, without any good results, and upon inquiry you find they have never seen the physician but once; that he gave them the prescription and told them to come back in a few days, but they did not think it was necessary to return as they were taking the medicine. Or you ask a patient who has been quite sick, why a physician was not called in earlier. "Well, doctor, our cook had some medicine a doctor gave her when she was suffering nearly the same way. It cured her in a few days, so I thought I would try it." Take any long continued disease, syphilis for instance, your patient will come regularly during the active stage, but as soon as the symptoms are in obeyance, his attendance will either cease or become irregular, though he may have his prescription filled and take it spasmodically, but when his symptoms return he never forgets to blame his physician for the bad results.

The remedy that suggests itself to our minds for the correction of this evil, is to have printed on all prescription blanks, *This prescription not to be refilled or a copy given.* At first blush this may seem arbitrary and uncalled for, and apparently means additional trouble and inconvenience for both physician and patient, but its advantages are many, as can be seen. It insures the physician seeing his patient, his condition, whether the remedies are having the desired effect or necessary changes should be made, and whether the directions as to dose, regularity of taking, etc., are carried out properly. These few words added to the prescription blanks means, more fees for the physician and quicker cure for the patient; you can keep your patient in regular attendance and under proper observation, for we can regulate the quantity for any desired length of time, and the patient necessarily returns to you before he can have it refilled. If they do not return, we have the satisfaction of knowing that they are not getting the benefit of one experience without paying for it, nor will it be possible for them to "hack" the prescription around promiscuously.

There is one class of prescriptions that by all means should have this on them—any prescription that contains morphine, chloral, whiskey or any drug that your patient is apt to fall into the habitual use of. A physician told me an incident that occurred in his practice, that fully illustrates how a patient may hold on to and take a prescription of this kind. "One day a man I did not recognize, but who was redolent with the fumes of whiskey, came up and spoke to me, Why, doctor, you don't recognize me?' 'No, I do not,' I replied. 'Well do you remember this?' he said, reaching into his pocket and pulling out a well worn piece of paper, which he handed me. 'It's a prescription you gave me about fifteen years ago and I have been taking it ever since, but I take lots more now than you told me to.' I read the paper, which was a prescription for whiskey I had given him. I

remembered the man, as a patient, who, while convalescent, I had told to get a bottle of whiskey and take three drinks a day until he was well. But he had replied that he was a prohibitionist and a church member—he could not unless I wrote him a prescription. And it was this he had and had continued the use of ever since, still drinking under the subtifuge of this piece of paper. "Well," the doctor said as he finished, "I tore up the prescription and told him he would have to get some one else's authority to continue." W. F. W.

